# Macrophage-derived CCL20–CCR6 signaling as a driver of immune recruitment in abdominal aortic aneurysm

**DOI:** 10.3389/fimmu.2026.1836038

**Published:** 2026-05-08

**Authors:** Cheng Wang, Ling Ye

**Affiliations:** 1 Department of Vascular Surgery, Mianyang Central Hospital, School of Medicine, University of Electronic Science and Technology of China, Mianyang, China; 2 Department of Neurosurgery, Mianyang Central Hospital, School of Medicine, University of Electronic Science and Technology of China, Mianyang, China

**Keywords:** abdominal aortic aneurysm, CCL20–CCR6 axis, immune microenvironment, inflammation, macrophages

## 1 Introduction

The absence of effective medical therapy for abdominal aortic aneurysm (AAA) remains a major clinical challenge, especially because the disease often progresses silently until catastrophic rupture. Against this background, mechanistic studies that clarify how inflammatory and immune pathways shape aneurysm biology are of considerable interest. In AAA, innate immune activation, maladaptive macrophage polarization, and subsequent recruitment of adaptive immune cells jointly contribute to extracellular matrix degradation, vascular smooth muscle cell loss, and progressive weakening of the aortic wall (1–5).

In this context, Ren et al. (6) provide a timely and multidimensional analysis of the macrophage-derived CCL20–CCR6 chemokine axis in AAA. Their central message is that imbalanced macrophage polarization favors CCL20 production, which in turn promotes recruitment of CCR6-expressing lymphocytes, especially T and B cells, thereby amplifying local inflammation and aneurysm progression. This work not only advances our understanding of immune-cell crosstalk in AAA, but also highlights CCL20–CCR6 signaling as a plausible biomarker-linked and therapeutically actionable pathway.

## 2 Immune microenvironment and AAA

In the immune microenvironment of AAA, the infiltration and functional imbalance of immune cells are key factors driving the structural destruction of the vessel wall and local inflammatory responses (7). Previous studies have demonstrated that macrophages play a central role in the immune response within AAA (8). Rather than acting solely as effector cells, macrophages also function as organizers of the inflammatory niche by regulating chemokine gradients and shaping adaptive immune recruitment. Ren et al. (6) used single-cell RNA sequencing to systematically reveal the polarization imbalance of macrophages in AAA tissues, with a predominance of M1 macrophages. This imbalance drives local immune responses and accelerates vascular wall destruction.

Immunohistochemistry further confirmed the significant increase in T cells and B cells in AAA tissues, which is closely associated with macrophage infiltration and polarization. Macrophages, by secreting CCL20, activate the CCR6 receptor, which facilitates the recruitment of T and B cells, thereby promoting the onset and progression of AAA. Accordingly, the study places macrophages at the center of a feed-forward inflammatory circuit, in which innate immune dysregulation is translated into sustained adaptive immune accumulation within the aneurysmal wall. This conceptual advance is important because it moves the field beyond descriptive immune infiltration and toward a more organized cellular communication model of AAA progression.

## 3 Mechanism of CCL20-CCR6 Axis in AAA

CCL20 is an important chemokine widely expressed in various cells of the vascular wall, including endothelial cells, smooth muscle cells, and macrophages (6). In the immune microenvironment of AAA, CCL20 is predominantly secreted by macrophages and recruits immune cells through its specific receptor, CCR6 (6). Studies have shown that macrophages use the CCL20-CCR6 axis to recruit T cells, B cells, and natural killer T cells to the AAA site, thereby exacerbating local inflammation and vascular wall damage (6).

Ren et al. (6) further validated this mechanism using experimental mouse models and clinical patient samples. In the AAA mouse model, blocking the CCL20-CCR6 axis significantly reduced immune cell infiltration and inhibited AAA progression. Additionally, Ren et al. (6) found that CCL20 was not only highly expressed in local tissues but also elevated in serum, correlating with the risk of AAA development. By analyzing data from the UK Biobank, the study further confirmed the potential of CCL20 as a biomarker for AAA progression.

One of the major strengths of this study is its integrative experimental design. The authors combine single-cell RNA sequencing, bulk RNA sequencing, human tissue validation, serum biomarker assessment, cell-cell communication inference, *in vitro* chemotaxis assays, and *in vivo* perturbation studies in a murine AAA model. This layered strategy strengthens the overall argument because it links transcriptional observations to histological validation, circulating biomarker data, functional migration assays, and disease modulation *in vivo*, rather than relying on any single analytical platform.

The translational significance of this work is also reinforced by prior biomarker literature. Earlier studies have suggested that circulating CCL20 may be elevated in AAA (9). In addition, recent large-scale plasma proteomic studies further support the broader relevance of circulating inflammatory mediators to vascular disease phenotypes and risk stratification (10). Although these population-level data do not by themselves establish mechanism, they strengthen the clinical plausibility of CCL20 as a measurable signal linked to aneurysm biology.

## 4 Strengths, interpretation, and limitations

This study offers valuable insights into the role of the CCL20-CCR6 axis in AAA progression, though several limitations remain. First, the relatively small sample size, with data primarily derived from a single-center study and animal models, limits the generalizability and applicability of the findings. Future research should focus on expanding sample sizes, particularly through large-scale, multicenter clinical studies, to validate the CCL20-CCR6 axis’s role across diverse patient populations. Additionally, integrating multi-omics approaches (e.g., genomics, transcriptomics, immunomics) will help better understand the immune mechanisms of this axis at different stages of AAA.

Secondly, while the study demonstrated that blocking the CCL20-CCR6 axis in animal models significantly inhibits AAA progression, translating these results into clinical practice, particularly regarding long-term efficacy and safety, requires further validation through extensive clinical trials. Effective treatment protocols and ensuring the safety of CCL20 or CCR6-targeting therapies remain critical challenges.

A further point that deserves emphasis is the analytical limitation of communication-inference approaches. CellChat analysis provides a useful hypothesis-generating framework for identifying candidate ligand-receptor interactions; however, such analyses remain inferential and are based primarily on transcript abundance rather than direct measurement of ligand secretion, receptor occupancy, or *in situ* signaling activity. Therefore, the proposed CCL20–CCR6 communication network should be interpreted as biologically plausible rather than definitively proven at the protein-function level. Additional spatially resolved proteomic, receptor-binding, or ex vivo functional studies would be valuable to validate whether this signaling axis is quantitatively dominant within human AAA lesions.

Moreover, although the murine perturbation experiments provide important mechanistic support, the Ang II-induced ApoE−/− model does not fully recapitulate the full biological heterogeneity of human AAA. The inflammatory milieu, temporal evolution, and cellular composition of experimental aneurysms may differ from those of human disease, and these differences should be considered when extrapolating therapeutic implications.

Furthermore, while the study focused on the CCL20-CCR6 axis, further investigation is needed into how this axis activates downstream signaling pathways, such as NF-κB, MAPK, and JAK/STAT, which may play crucial roles in immune cell polarization and inflammation in AAA. Equally important, future studies should clarify whether CCL20 is merely a marker of an activated inflammatory niche or whether it acts as a non-redundant upstream driver whose blockade would remain effective in the presence of other chemokine programs.

## 5 Conclusion

Ren et al. identify the CCL20–CCR6 axis as a plausible macrophage-centered mechanism linking innate immune dysregulation to lymphocyte recruitment in AAA. By integrating transcriptomic, histological, serum, and functional evidence, the study provides meaningful insight into how immune-cell communication may contribute to aneurysm progression. Although further validation is required, particularly in relation to causal signaling and clinical translation, this work highlights CCL20–CCR6 signaling as a promising direction for biomarker development and mechanism-based therapeutic exploration in AAA.
